# Drivers of antimicrobial resistance within the communities of Nepal from One Health perspective: a scoping review

**DOI:** 10.3389/fpubh.2024.1384779

**Published:** 2024-04-19

**Authors:** Ayuska Parajuli, Jessica Mitchell, Natalie King, Abriti Arjyal, Sophia Latham, Rebecca King, Sushil Baral

**Affiliations:** ^1^HERD International, Lalitpur, Nepal; ^2^Nuffield Centre for International Health and Development, University of Leeds, Leeds, United Kingdom; ^3^Academic Unit of Health Economics, University of Leeds, Leeds, United Kingdom; ^4^Institute of Infection, Veterinary and Ecological Sciences, University of Liverpool, Liverpool, United Kingdom

**Keywords:** antimicrobial resistance, community, Nepal, drivers and barriers, One Health

## Abstract

**Background:**

A major driver of antimicrobial resistance (AMR) is the inappropriate use of antimicrobials. At the community level, people are often engaged in behaviors that drive AMR within human, animal, and environmental (One Health) impacts. This scoping review consolidates research to determine (a) the community’s knowledge, attitudes, and practices around AMR; (b) existing community-based interventions; and (c) barriers and enablers to addressing AMR in Nepal.

**Methods:**

This scoping review follows the Joanna Briggs Institute scoping review methodology. Literature indexed in PubMed, Scopus, CINAHL, Global Index Medicus, HINARI-SUMMON, Embase (Ovid), Global Health (Ovid), CAB Abstracts (Ovid), Web of Science, and Google Scholar between January 2000 and January 2023 were reviewed for inclusion. Articles were included in the review if they considered the issues of AMR at the community level in Nepal; this excluded clinical and laboratory-based studies. A total of 47 studies met these criteria, were extracted, and analyzed to consolidate the key themes.

**Results:**

A total of 31 (66%) articles exclusively included human health; five (11%) concentrated only on animal health; no studies solely focused on environmental aspects of AMR; and the remaining studies jointly presented human, animal, and environmental aspects. Findings revealed inadequate knowledge accompanied by inappropriate practice in both the human and animal health sectors. Four community interventions improved knowledge and practices on the appropriate use of antimicrobials among community people. However, various social and economic factors were found as barriers to the appropriate use of antimicrobials in the community.

**Conclusion:**

Community engagement and One Health approaches could be key tools to improve awareness of AMR and promote behavioral change related to AM use in communities, as current studies have revealed inadequate knowledge alongside inappropriate practices shared in both human and animal health sectors.

**Systematic review registration:**

DOI: 10.17605/OSF.IO/FV326

## 1 Introduction

Antimicrobial resistance (AMR) is one of the most urgent global health threats of the Anthropocene (1). In 2019, bacterial AMR alone was shown to be directly responsible for almost 1.3 million global human deaths (2). The full annual costs of AMR are likely to be considerably higher once human deaths attributed to resistant viral, fungal, and pathogenic infections are included, plus the deaths of animals due to resistant microbes (3–6). Although the evolution of resistance is a natural process, the key driver of AMR is the injudicious use of antimicrobial drugs (1, 7, 8). This includes using the wrong drug for the wrong illness, failure to complete a full course or dose of the right drug, prophylactic use of antimicrobials, or using antimicrobials as growth promoters (7, 9–11). Antimicrobial disposal is also a primary driver of AMR, as across the world, environments such as rivers, soils, and even snow are becoming contaminated by antimicrobial waste, which in turn exposes many more microbes to drive the evolution of resistance (10, 12–14). In turn, the movement of resistant microbes and antimicrobial waste can facilitate the spread of AMR to diverse populations of humans and animals far from the original source of contamination, such as hospitals, farms, soil, and water. The human-animal-environmental health impacts of AMR mean it is often described as a One Health problem, yet human behavior at the systemic, organizational, and individual level is responsible for many AMR drivers (15, 16).

AMR is a particular challenge for low- and middle-income countries (LMICs) with the greatest burden of human AMR-associated deaths occurring within western sub-Saharan Africa and then South Asia (2). Such trends can again, primarily, be explained by the complex and contextually specific behavioral drivers of AMR within LMICs. For example, the existence of pluralistic healthcare systems such as pharmacies, clinics, private hospitals, and government health facilities plays an important role in causing AMR (17–19). This leads to no access to antimicrobials for some people at all, while others are over-prescribed antimicrobials for low-risk infections, and others are inappropriately given antimicrobials, for example, antibiotics to treat viral infections (17–19). Population growth and resulting demand for food have dramatically increased the use of antimicrobials in food-producing animals and crops in LMICs (11, 20, 21). Although policymakers are attempting to curb the use of antimicrobials as growth promoters or prophylaxes, agricultural products are often poorly regulated in terms of their antimicrobial content, and drugs are frequently added to feed and fertilizers at the point of sale (11, 21). LMICs also have less well-developed water, sanitation, and hygiene infrastructure, which means infections are more common in general while also increasing the likelihood of resistant infections evolving and spreading. The interaction of these weak infrastructures with climatic fluctuations such as drought and monsoon seasons, which are usually followed by epidemics of infectious disease, can also create temporal hotspots for the evolution and spread of AMR (18).

Nepal experiences many of these common challenges especially regarding the provision of antimicrobial medicines from a qualified health or veterinary professional (22, 23). Many rural people do not have easy access to formally trained providers which is leading to high reliance on over-the-counter (OTC) drug sellers and non-prescription provision of antimicrobials (17, 24, 25). Animal health provisions are limited in both rural and urban areas, meaning antimicrobials are often sourced from OTC and are regularly used without veterinary supervision or guidance, including as feed supplements and growth promoters (22). There is also a lack of laboratory facilities to provide diagnostic services for human, veterinary, and environmental samples. This refers to the diagnosis, provision, or prescribing of antimicrobials, which is often done based on symptomatic assessment only (22, 26, 27). Nepal also faces specific issues relating to the supply of medical products including antimicrobials to government health facilities across the country (28). Many cannot always provide a full course of antimicrobial drugs, meaning the responsibility is on individuals to return to their provider to complete the course (19, 24, 28). Due to high out-of-pocket expenditure of health services, traditional healers, informal providers, and OTC drug sellers play an important role in community and veterinary healthcare (19, 24, 28).

Nepal has made great strides in governance around AMR, and the recently completed National AMR Action Plan (known as a NAP) is about to be endorsed at the federal level (29). The new NAP includes commitments to addressing AMR via increased One Health laboratory capacity, strengthening health and veterinary systems, prescriber training, and global governance and collaboration (29). It also includes a generous component regarding public and community-level engagement with the issue of AMR via outreach, awareness-raising activities, and education. The concept of public engagement in AMR is incredibly important, specifically in a country like Nepal where communities are familiar with, and often reliant on OTC drug access. Any changes to the supply side of antimicrobials are unlikely to be successful without community-level buy-in (25, 28, 30). Hence, the concept of Community engagement (CE) should be emphasized around AMR in Nepal. CE goes beyond simple awareness-raising tactics such as poster campaigns and educational flyers to exchange knowledge with people regarding why they engage in AMR-driving behaviors and what could be done to alter these behaviors. The global literature on CE and AMR suggests that this approach is more successful at creating sustained behavior change than awareness-raising activities alone (31).

Over the past twenty years, a growing body of literature has been published on the specific topic of antibiotic misuse with the majority centered within the human health sector and limited focus on community engagement (16, 19, 22, 24, 28, 32). However, the growing scope of this research is yet to be synthesized to provide a comprehensive overview of community-level AMR dynamics from a One Health perspective. The objective of this scoping review is to map existing research on the One Health drivers of AMR and existing enablers and barriers to addressing AMR at the community level in Nepal. To do so, we will consider four specific research questions:

1. What are the knowledge, attitudes, and practices on appropriate use of antimicrobials within the communities of Nepal?
2. What are the existing interventions to address AMR in the community settings in Nepal?
3. What are the current enablers and barriers to address drivers of AMR in community settings in Nepal?

Based on available data, we will explore potential differences linked to gender, geographic location (including rural/urban), ethnicity, and education status of the community people across all research questions. Thus, we anticipate that the findings will generate nationally meaningful recommendations for AMR policymakers that consider the One Health dynamics of AMR from a community level perspective.

## 2 Methods

### 2.1 Protocol and registration

This scoping review was designed to identify the drivers of antimicrobial resistance in Nepal from a One Health perspective, in accordance with the Joanna Briggs Institute (JBI) manual and the Preferred Reporting Items for Systematic Reviews and Meta-Analysis extension for Scoping Review (PRISMA-ScR) checklist (33, 34). A protocol was developed and registered on the Open Science Framework (OSF) and can be accessed here via the DOI: 10.17605/OSF.IO/FV326 (35).

### 2.2 Eligibility criteria

This review includes research addressing the knowledge, attitudes, and practice of antimicrobial use among lay persons, i.e., a person without formal background/training on AMR, including patients of hospital and out-patient departments, from the One Health perspective (human, animal, and environmental health). Community pharmacies hold a significant role in shaping antimicrobial usage within communities. Therefore, the review includes article related to OTC drug use and the dispensing practices of pharmacies serving communities. Various types of published peer-reviewed articles, regardless of their design—be it original research, secondary analysis, reviews, editorials, perspectives, commentaries, discussions, or letters to the editor—were incorporated.

The inclusion criteria involved studies conducted in Nepal or utilizing data from Nepal exclusively, while omitting laboratory-based and clinical research. Additionally, studies focusing on the knowledge, attitudes, and practices of individuals formally trained in AMR (e.g., community health workers, healthcare professionals, and medical/nursing students) were excluded.

### 2.3 Search strategy

The first author (AP) conducted a preliminary search in PubMed and Google Scholar to identify key terms used within the titles and abstracts of relevant articles. Following this, key search terms were developed iteratively by the authorship team, and a search strategy using individual database subject headings and free text words was constructed around two concepts: antimicrobials or drug resistance and Nepal terms. Full details of the search strategies and search authors can be found in the Supplementary material (S1). The database searches were conducted by two researchers (AP and NK) based on their institutional access, and all update searches were rerun by NK. All searches were peer-reviewed using the PRESS Checklist (36).

### 2.4 Information sources

Peer-reviewed articles were searched in the following 10 databases: PubMed, Scopus, CINAHL, Global Index Medicus, HINARI-SUMMON, Embase (Ovid), Global Health (Ovid), CAB Abstracts (Ovid), Web of Science, and Google Scholar. Google Scholar was searched using Publish or Perish software (37). We initiated searches from 25 November to 7 December 2023 and then updated the searches from 27 February to 28 February 2023. Details on searches are presented in the Supplementary material (S1).

Updated search (secondary search) did not include searches conducted on HINARI-SUMMON due to the coverage overlaps. It was discovered that the content from the HINARI-SUMMON significantly overlapped with other databases’ content. Hence, to avoid redundancy and maximize efficiency, research team chose to exclude this overlapping database.

Searches were limited to studies published beyond 2000 because this was the point at which One Health-style AMR surveillance (for human and animal pathogens) began in Nepal (24).

### 2.5 Selection of sources of evidence

The references were deduplicated in the bibliographic management software, EndNote version 20, and transferred into the Covidence collaboration platform (38). Two authors (AP and SP for the initial search and AP and JM for the secondary search) independently screened by title and abstracts and carried out the full text review for the eligibility of articles. Any conflicts raised between the reviewers during screening were resolved by discussion with a third author (JM for the initial search and AA for the secondary search).

### 2.6 Data charting process

A data extraction template (Supplementary material, S2) was developed with the details of information to be recorded on the Covidence number of the article, author, published year, title of the article, journal name, type of article, objectives, study design, methods, interventions (if any), study population, sample size, sampling technique, period of data collection, and place where the study was conducted.

Data were extracted according to four major themes, i.e., human health, animal health, agriculture, and environment. Two authors (AP and JM) independently piloted a draft data extraction template on five studies to ensure that all relevant information was retrieved. Charting of the data was an iterative process during the initial stages of data extraction, and the charting template was updated into further subthemes for human and animal health. Human health was further divided into subthemes, namely, sources and dispensing practice of antibiotics, knowledge, and attitude (on self-medication, antimicrobial use, and antimicrobial resistance), practice of using antimicrobials, use of antibiotics among children (under 10 years), factors affecting knowledge, attitude, and practice of antimicrobial, drivers of care seeking and antibiotic selling at drug shops, and reasons for non-adherence toward antimicrobials.

Subthemes identified under animal health were animal husbandry practices, sources, and dispensing practice of antimicrobial, practice of using antimicrobial, and knowledge on antimicrobial and AMR. Data charting was done by a single reviewer (AP), and any confusions raised were discussed and clarified with the broader review team (AA, JM, NK, and SP).

## 3 Results

**Figure 1:**
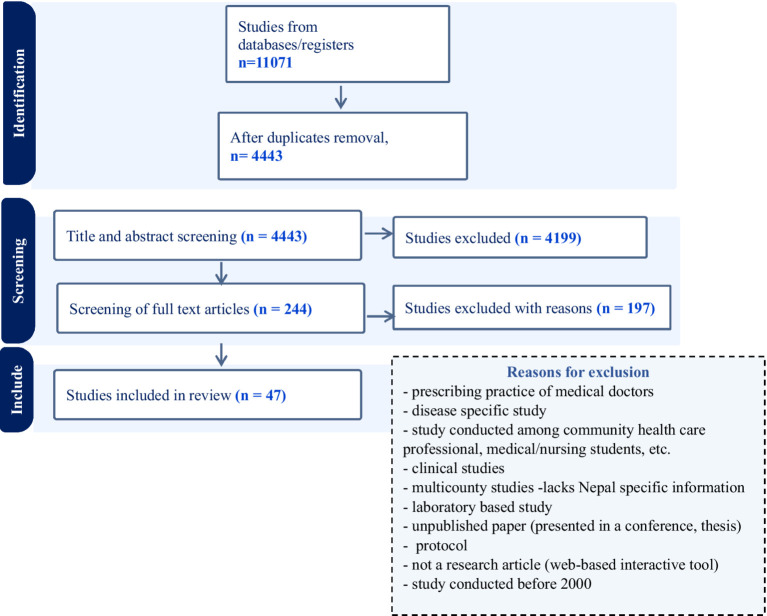
Flow diagram of the study selection process (PRISMA-ScR).

The original search identified 11,071 items of which 6,628 duplicates were removed and 4,443 progressed to title and abstract screening. Of these, 244 progressed to full text screening against the inclusion criteria. This process resulted in final dataset of 47 items. Figure 1 summarizes the screening and data extraction process for the search items according to the PRISMA-ScR (34). The PRISMA-ScR checklist used in this study can be found in the Supplementary material (S3).

### 3.1 Establishing terminological clarity in results

In this study, we have defined *community people* as general public or ordinary person who are non-health professional and have not received any kind of formal training on AMR (e.g., community health worker, healthcare professional, and medical/nursing students).

The included studies have presented a range of community people. They are school and university students, schoolteachers, family members of school students, community household members (both adults and children), small- and large-scale poultry farmers, poultry farm owners, farmers, patients attending pharmacies and hospitals, outpatients of hospitals, mothers of pediatric children, community-level policymakers, and community people.

In addition, this scoping review includes the dispensing practices of drug sellers in the community to unfold various intertwined factors influencing community people’s behavior contributing to AMR. The term *drug sellers* in this study refer to community pharmacy personnel, owners and managers of pharmacy, medicals, and agrovets. Here, *pharmacy* refers to a store where medications, drugs, and pharmaceutical products are dispensed or sold. Similarly, *medicals* are drug shop-based practitioners involved in treatment services of people, which may or may not have the provision of a physician. Sometimes, it is established as medicals, whereas in some instances, pharmacies turn out to be medical. Similarly, *agrovet* refers to a facility within agriculture that focuses on providing veterinary products, services, and expertise specifically tailored for livestock and farm animals. Pharmacy, medical, and agrovets are ideally supposed to be operated by trained paramedics. However, sometimes they are operated by untrained ordinary people who are family members and friends of trained paramedics.

Lastly, different types of drugs used to treat microbial infections are called *antimicrobials*, which include antibacterial/antibiotic, antiviral, antifungal, and antiparasitic. The included studies have used both the terms, *antibiotic* (28, 39–53) and *antimicrobial* (16, 19, 25, 26, 32, 54–65). In some studies, antibiotic terminology has been used to explore the practices of community, whereas antimicrobials and antimicrobial resistance words have been used to describe the concept (24, 66–71).

### 3.2 Description of included articles

A total of 47 articles were included in this review. Of these, 36 (77%) were original research articles, five (11%) were letters to the editor, and there were one each of the following article formats: editorial, review, viewpoint, opinion piece, short communication and secondary analysis. Of the 36 original articles, the majority (26, 72%) were cross-sectional studies, while two (6%) were case–controlled studies, four (11%) were interventional, and four (11%) used qualitative approaches such as focus group discussions (FGDs) or interviews (Table 1).

**Table 1:** Characteristics of the included articles.

Title	Study design/type of article	Methods	One Health sphere	Study population	Study site	Province	Ecological zone
Acharya and Wilson (63)	Review	Review	One Health	NA	NA	NA	NA
Acharya et al. (26)	Letter to the editor	NA	One Health	NA	Nepal	NA	NA
Acharya et al. (48)	Cross-sectional	Qualitative and quantitative	Human	Pharmacy owners and managers	Kathmandu, Bhaktapur, Lalitpur Kavrepalanchok and Dhading	Bagmati	Hilly region of the valley
Acharya (61)	Letter to the editor	NA	One Health	NA	Nepal	NA	NA
Acharya et al. (70)	Letter to the editor		One Health	NA	Nepal	NA	NA
Adhikari et al. (19)	Qualitative	Interview/Focused group discussion	Human	Patients, clinicians and drug sellers located around hospitals	Sunsari, Lalitpur, Rupandehi	Koshi, Bagmati, Lumbini	Hilly, terai mountain
Ansari (41)	Cross-sectional	Quantitative	Human	Community pharmacies	Bara and Parsa	Madhesh	Terai
Bam et al. (72)	Cross-sectional	Quantitative	Human	TB Patients	Kathmandu	Bagmati	Hilly region of the valley
Ban et al. (66)	Cross-sectional	Quantitative	Human	Medicine shop-based practitioners & physician-run clinics	25 districts of Nepal	All 7 province	Hilly, terai, mountain
Chapagain and Rauniyar (73)	Cross-sectional	Quantitative	Human	Community households	Dharan metropolitan city, Sunsari	Koshi	Terai
Deo et al. (52)	Cross-sectional	Quantitative	Human	School Students	Kathmandu	Bagmati	Hilly region of the valley
Dhakal and Gompo (71)	Cross-sectional	Quantitative	Animal	Farmers	Kathmandu and Chitwan districts	Bagmati	Hilly region of the valley, terai
Goswami et al. (51)	Cross-sectional	Quantitative	Human	Community pharmacy personnel, irrespective of their qualifications	Jhapa, Morang, Sunsari	Koshi	Terai
Holloway and Gautam (74)	Cross-sectional	Quantitative	Human	Outpatients	Bhojpur, Taplejung and Panchthar	Koshi	Hilly
Holloway et al. (49)	Interventional	Quantitative	Human	Community households	Khotang, Panchthar, Sankhuwasabha and Taplejung	Koshi	Hilly
Jones et al. (32)	Qualitative	Observation/interview/focus group	Human	Members of the community	Kathmandu and Bhaktapur	Bagmati	Hilly region of the valley
Kafle et al. (47)	Interventional	Quantitative	Human	School teacher, school students their family members, and journalists	Bhaktapur District	Bagmati	Hilly region of the valley
Khadka et al. (58)	Cross-sectional	Quantitative	Human	Outpatients attending tertiary hospital, community people, their care takers, visitors and other contacts (Community people)	Kathmandu	Bagmati	Hilly region of the valley
Khan and Miya (44)	Interventional	Qualitative and quantitative	Human	Guardians of Pediatric Patients	Pokhara valley	Gandaki	Hilly region of the valley
Koirala et al. (46)	Cross-sectional	Quantitative	Animal	Broiler farmers with flock size greater than 3,000	Kathmandu Valley (Kathmandu, Bhaktapur, Lalitpur)	Bagmati	Hilly region of the valley
Koju et al. (60)	Cross-sectional: Mixed method	Qualitative and quantitative	Animal	Animal Husbandry Stakeholders	Dhulikhel Municipality, Kavrepalanchok	Bagmati	Hilly
Rijal et al. (24)	Cross-sectional	Quantitative	One Health	Clinicians, patients, private drug sellers, livestock and poultry farmers, diagnostic laboratory staff	8 districts	All 7 province	Hilly region of the valley, terai
Acharya and Subedi (68)	Opinion piece	NA	Human	NA	Nepal	NA	NA
Acharya et al. (54)	Letter to the editor	NA	One Health	NA	Nepal	NA	NA
Lambrou et al. (55)	Cross-sectional	Quantitative	Animal	Poultry farmers with flocks of >100 chickens, ducks, or turkeys.	Chitwan	Lumbini	Terai
Mishra et al. (75)	Case controlled	Quantitative	Human	TB Patients	Kaski	Gandaki	Hilly region of the valley
Mishra et al. (76)	Case controlled	Quantitative	Human	Outpatients	Kaski	Gandaki	Hilly region of the valley
Mishra et al. (50)	Cross-sectional	Quantitative	Human	Community household (Household heads)	Rolpa	Lumbini	Hilly, mountain
Mitchell et al. (16)	Qualitative	Qualitative study: Thematic analysis	One Health	Community people	Kathmandu	Bagmati	Hilly region of the valley
Nelson et al. (65)	Cross-sectional	Qualitative and quantitative	Animal	Farm owners of poultry farm	Chitwan	Bagmati	Terai
Nepal et al. (40)	Cross-sectional	Quantitative	Human	Community household; head of household or most senior member of household	Kapilvastu	Lumbini	Terai
Nepal et al. (69)	Cross-sectional	Quantitative	Human	Patients attending private pharmacies	Rupandehi	Lumbini	Terai
Nepal et al. (28)	Qualitative	Interview/Focused group discussion	Human	Service providers and policymakers	Rupandehi	Lumbini	Terai
Ng et al. (77)	Interventional	Quantitative	Animal	Community households	Chitwan	Bagmati	Terai
Paudel and Aryal (78)	Cross-sectional	Quantitative	Human	Patients	Pokhara valley	Gandaki	Hilly region of the valley
Pokharel and Adhikari (25)	viewpoints	NA	Human	NA	Nepal	NA	NA
Pokharel and Adhikari (64)	Editorial	NA	One Health	NA	LMIC (including Nepal)	NA	NA
Poudel et al. (67)	Letter to the editor	NA	Human	NA	Nepal	NA	NA
Rogawski et al.(43)	Cross-sectional	Quantitative	Human	Mothers of children <2 years	8 countries including Nepal (Bhaktapur)	Bagmati	Hilly region of the valley
Shah et al. (53)	Cross-sectional	Quantitative	Human	University students	Kathmandu	Bagmati	Hilly region of the valley
Shankar et al. (62)	Cross-sectional	Quantitative	Human	Community households	Pokhara valley	Gandaki	Hilly, mountains
Shrestha et al. (57)	Cross-sectional	Mixed methods	Human	Members of the community	Dhulikhel municipality (Kavrepalanchok)	Bagmati	Hilly
Shrestha et al. (56)	Cross-sectional	Quantitative	Human	Pharmacy employees	Lalitpur	Bagmati	Hilly region of the valley
Shrestha et al. (45)	Cross-sectional	Quantitative	Human	General Public	Kathmandu	Bagmati	Hilly region of the valley
Vaidya et al. (42)	Cross-sectional	Quantitative	Human	Outpatients	Kathmandu and Kavrepalanchok	Bagmati	Hilly region of the valley
Yadav et al. (59)	Short Communication	Short Communication	One Health	NA	Nepal	NA	NA
Zheng et al. (39)	Retrospective Study/Secondary data analysis	Quantitative	Human	Children < five years old	Nepal	All 7 provinces	Hilly, terai, mountain

Studies are spread across the provinces of Nepal. However, study sites are not applicable in the case of letters to the editor, editorial, review, viewpoint, opinion piece, and short communication format of article. Of 37 studies (36 original articles and 1 secondary analysis), three (8%) focused their work across seven provinces, and one (3%) study was conducted in three provinces, whereas the remaining studies focused on specific provinces, i.e., Koshi (4, 11%), Madhesh (1, 3%), Bagmati (18, 49%), Gandaki (5, 13%), and Lumbini (5, 13%) provinces. This also represents geographical spread across different geo-ecological regions of Nepal, namely terai, hilly, and mountain. The distribution of study as per the geo-ecological regions are hilly only (4), terai only (9), hilly region of the valley (17), terai and hilly region of the valley (2), hilly and mountain, (2) and hilly, terai, and mountain (3) (Table 1).

Similarly, of the 47 included studies, 31 (66%) articles exclusively included the human health aspects of AMR, while five (11%) concentrated only on AMR in animal health. No studies have solely focused on the environmental aspects of AMR. Seven (15%) studies focused on both human and animal health aspects of AMR. Similarly, four (8%) studies slightly mentioned about the environmental aspect of AMR, primarily emphasizing human and animal health. Characteristics of all the articles included in the studies are presented in Table 1.

### 3.3 Prevalent themes and emerging patterns

Studies highlighted limited knowledge on antimicrobials and AMR among diverse community populations including community people, students, drug suppliers (human and animal health), farmers, and patients and their role in contributing to the development of AMR. Within this context, studies exploring the practices of using antimicrobials in both the human and animal health sectors highlighted the growing prevalence of inappropriate use of antimicrobials among community people and drug suppliers (Table 2).

**Table 2:** Themes identified during the review.

Themes identified	Human	Animal	Environment
Knowledge and attitude on antimicrobials and AMR	(19, 24–26, 28, 40, 44, 45, 47, 50–52, 54, 56–59, 68)	(19, 24, 26, 54, 55, 59, 60, 63, 71)	(24)
Practice of using antimicrobials in human health	(19, 24–26, 28, 32, 39–44, 48, 50, 53, 55–64, 66, 67, 69, 70, 72, 73, 75, 76, 78)		
Practice of using antimicrobials in animal health		(26, 46, 55, 59, 60, 63, 65, 70, 71, 77)	
Barriers to address drivers of AMR	(19, 24–26, 28, 40, 42, 44, 48, 51, 52, 56, 57, 59, 60, 62–64, 67, 69, 72, 74–77)	(26, 60, 77)	
Enablers to address driver of AMR	(16, 32, 44, 47, 49, 54, 56, 59, 68)	(54, 77)	(54)

Furthermore, studies note that various factors acted as barriers to address the drivers of AMR in the community for both human and animal health sectors. However, different types of community-based interventions with a range of approaches used are shown to lead to positive AMR-related outcomes across various settings and populations. This section delineates and analyzes these prevalent themes while elucidating their implications for future research directions and policy considerations (Table 2).

### 3.4 Knowledge and attitude on antimicrobials and AMR: human and animal health

Knowledge on antibiotics, antibiotic resistance (ABR), and AMR was generally inadequate among community people (24, 40, 57), patients (19, 28), students (52), livestock farmers (71), commercial poultry producers (55, 71), pharmacy and medical (19, 51), and agrovets (19, 54, 60). Community people and small-scale poultry farmers were not able to identify antibiotics even though they had used it (40, 71). However, farmers engaged in large-scale farming were able to identify antibiotics (55). Commercial poultry farmers (55, 60, 71) and different members of the community lacked awareness on the prudent use of antimicrobials to increase the effectiveness of antibiotics for a longer period of time (24, 28, 56, 59, 63) (Table 3).

**Table 3:** Knowledge, attitude, and practice on antimicrobials and AMR.

Key findings		Community		Pharmacy, medical, and agrovets	
		Human	Animal	Human	Animal
Knowledge and attitude	Knowledge on antibiotics and antimicrobial resistance	(19, 24, 26, 28, 40, 45, 47, 52, 54, 57, 68)	(24, 26, 54, 55, 71)	(19, 24, 51, 54, 56)	(19, 54, 60)
	Appropriate use of antibiotics	(24–26, 28, 40, 44, 47, 57–59)	(24, 26, 55, 59, 60, 71)	(19, 56)	(19, 60, 63)
	Factors influencing knowledge/education	(24, 40, 44, 45, 50)	(55)	(41, 51)	
Practice	Common places to receive antimicrobials	(19, 24, 25, 28, 39, 42, 56, 57, 62–64, 69, 73)	(55)	(55)	
	Practice of using antimicrobials	(19, 24, 26, 28, 32, 40, 42, 48, 50, 53, 56–59, 62–64, 67, 69, 70, 73, 78)	(26, 46, 55, 59, 60, 63, 70, 71)	(19, 24–26, 28, 41, 48, 56, 57, 61, 63, 67, 69, 70)	(26, 60, 70)
	Practice of using antimicrobials among under 0- to 12-year-old children	(39, 42–44, 48)		(66)	
	Factors influencing practice of using antimicrobial	(28, 39, 44, 57, 58, 67, 72, 75, 76)	(60)	(41, 60, 69)	
	Self-medication	(24, 25, 42, 45, 50, 53, 57, 62, 63, 73, 78)			
	Animal husbandry practice		(26, 63, 65, 71, 77)		
	Poor infection prevention	(26, 63, 65)	(26, 63, 65)	(26, 70)	
	Inappropriate waste disposal	(26, 63)		(26, 70)	

Antibiotics were perceived to be used to cure different illnesses such as the common cold, sore throat, headache, viral disease, skin infections/wound, and general weakness (28, 40, 63, 70) and could be discontinued once they started feeling better (24, 57). Nonetheless, a study conducted in seven districts of Nepal found that participants were aware of the necessity for farmers to limit antibiotic usage, improve infection prevention measures such as hand hygiene practices, and ensure up-to-date vaccinations for children (24) (Table 3).

On the other hand, reviewed studies revealed individuals in the community express dissatisfaction with a doctor’s visit if they did not receive antibiotics and would prefer seeking care from another doctor, driven by the belief that antibiotics can contribute to quicker recovery irrespective of its need to be used (28, 40, 48). However, in different studies conducted, individuals in the community were unsure if they could play an important role in preventing AMR and ABR by correctly using and not skipping the dose of antibiotics (19, 24, 28, 58) (Table 3).

### 3.5 Practice of using antimicrobial in human health

Many studies indicate that the first point of contact for community people during an illness is pharmacy, medical, followed by a hospital or clinic (28, 39, 56, 57, 63, 73). Their place of visit was determined by the perceived seriousness of their condition, and many described visiting drug sellers for mild-to-moderate illnesses (28, 39, 56, 57, 63, 73). A study found patients requested particular antibiotics from the drug shops providing prompts, such as showing the empty blisters or bottles and previous prescriptions used by themselves or family members (19). The practice of self-medication with antibiotics (without visiting the doctors and without any prescription for the current illness) was reported irrespective of the types of population (community people and college students), area of residence (urban/rural), sex, and age (24, 25, 42, 45, 50, 53, 57, 62, 63, 73, 78) (Table 3).

Other common community behaviors were failure to complete the full dose of antimicrobials and storing the leftover medicines for future and emergency situation (19, 57, 63). Reasons behind all these actions were saving time, previous experiences of being cured, and financial constraints of visiting formal health facilities (24, 32, 45, 50, 57, 62, 73). This review suggests there are some demographic and contextual trends in community-level antimicrobial practices. For example, the secondary analysis of Demographic and Health Survey (DHS) data (2006–2016) showed increased trend of using antibiotics in rural areas in comparison with urban for acute respiratory infection (ARI) and fever among children less than 5 years (39). One of the studies reported that as a child’s age advanced, the percentage of child taken to the hospital reduced, while the percentage of child taken to drugstore inclined (42). Community people as well as health worker at formal health facility reported community’s habit of taking antibiotics prior to seeking care (42, 59) (Table 3).

The literature reviewed suggested age, gender, area of residence (urban/rural), education, income, employment status, and occupation of an individual were significantly associated with knowledge, attitude, and practices on antibiotics and antibiotic resistance among community people (24, 40, 44, 45, 50). On the contrary, the appropriate dispensing practice of drug sellers was significantly associated with the number of years of experience, age, education, and registration status of pharmacist/medical (41, 51). Similarly, secondary analysis of DHS (2006–2016) showed an association between reduced use of antibiotics for diarrhea among children and improved toilet sanitation (39) (Table 3).

### 3.6 Practice of using antimicrobial in animal health

The use of antimicrobials in animal health presents similar practices to those encountered in human health, as veterinary drugs are sold without prescription, based on farmers’ demand and sellers’ self-prescription (26, 55, 63, 65). Major sources of veterinary drugs including antimicrobials were found to be agrovet and feed suppliers in the local community (26, 55, 63, 65). Reviewed literature found that farmers usually administer antibiotics to their animals relying on their previous experience and in consultation with neighbors (26, 55, 63). Antimicrobials were commonly used by the community people for different purposes, including disease prevention, treatment, growth promotion, and reduction in mortality of animals such as cattle, pigs, and poultry (26, 46, 55, 63, 70, 71). In a farmer-focused survey, the majority of the large-scale producers had knowledge about withdrawal periods; however, only some of them complied with them (55, 59, 60, 63) (Table 3).

Animal husbandry practices are key facet of the animal health sector associated with AMR from the perspective of infection prevention. Studies unveiled farmers’ limited knowledge and practices on biosecurity measures and good animal husbandry practices, such as the habit of disinfecting their foot covers before entering the farms, the arrangement of separate quarantine rooms, cleaning utensils before feeding birds, and disposing of used poultry litters far from the shed (63, 65, 71) (Table 3).

### 3.7 Practice: environmental perspective

Limited infection control methods, poor hospital waste management, improper waste segregation, disposal systems, medical waste processing, and poor sanitation and hygiene were identified as drivers of AMR from an environmental perspective (26, 54, 63, 70) (Table 3).

### 3.8 Barriers to address drivers of AMR: human and animal health

Literature reviewed suggested that a low physician-to-patient ratio (1:1724) had compelled community people to rely on pharmacists, medicals, and paramedics for their healthcare (42, 56, 64). Community-level barriers to seeking healthcare from formal health facilities included opportunity costs, time, low economic status, high out-of-pocket expenditure, long distance (rural locations), and gaps in doctor-patient relationships (19, 25, 28, 57, 62, 63, 67). Additionally, varying practices of buying and selling antimicrobials without prescription in the community reflected different aspects of existing barriers to address AMR in Nepal. Major barriers highlighted in the literature were limited knowledge on appropriate use of antimicrobials and AMR, as well as social and economic barriers perceived from both the community’s and the drug seller’s perspectives (19, 24, 26, 28, 40, 44, 48, 51, 52, 57, 59, 60, 64, 69, 75, 77). Studies revealed differences in pattern of purchasing antibiotics among those who have heard and not heard about antibiotics (Table 4).

**Table 4:** Barriers and enablers to address drivers of AMR.

Key findings		Human	Animal	Environment
Barriers for appropriate use of antimicrobial	COMMUNITY’S PERSPECTIVE			
	Knowledge and attitude on antimicrobial and AMR	(26, 28, 40, 52, 57, 59)	(26, 77)	
	Social aspect	(19, 48)		
	Economic aspect	(19, 28, 44, 57)	(60)	
	DRUG SELLER’S (pharmacy, medical, agrovets) PERSPECTIVE			
	Knowledge and attitude on antimicrobial and AMR	(19, 24, 51, 69)	(60)	
	Social aspect	(60)		
	Economic aspect	(19, 28, 48, 64, 75)		
	Communication between community people and drug seller/health worker	(72, 76)		
	Limited resources in government health facility	(28)		
Enablers for appropriate use of antimicrobial	Community interventions to improve knowledge or appropriate use of antimicrobial.	(16, 32, 44, 47, 49, 54, 56, 59)	(54, 77)	(54)
	Use of digital technology in improving knowledge	(54, 68)	(54)	(54)

Community people’s (those who have heard of antibiotics) demand for antibiotic was described as a social practice because this was driven by their past experiences of being cured from that specific medicine, and this behavior was inherited through the generations in urban, peri urban, and rural settings (19, 48). Dispensing antibiotic on people’s demand rather than only complying for the prescription-based dispensing of antibiotics was related to the economy of the drug sellers, where they feared losing the client as the client would get the desired medicine from another drug shop in the case of refusal (19, 28, 48, 64). On the other hand, people who did not know the term antibiotic, explain their symptoms and trust the drug sellers to decide on medicines to cure their illness (19, 48, 60). Additionally, profit-oriented drug sellers provided too many broad-spectrum and high-dose antibiotics at the same time, calling it a double-edged sword to cure presumptive diagnosis (19, 48, 64). Such interaction strengthened the social relation between community and dispensers, leading to repetitive visits (19, 48, 60). Hence, studies exposed entangled relationship between social and economic barriers seen in the community’s and drug sellers’ behavior (19, 48, 60, 64) (Table 4).

Furthermore, drug sellers’ limited knowledge on the appropriate use of antimicrobials and AMR was associated with the practice of dispensing incorrect doses and providing incorrect guidance or no advice on the appropriate intake of antimicrobials to the patients (19, 24, 51, 60, 69). Poor communication between patients and dispensers was significantly associated with non-adherence to medicines including antimicrobials (72, 76). Furthermore, people with less knowledge on AMR, who are non-literate, have low education, are unemployed, and are facing financial difficulties in treatment were found to have non-adherence to antimicrobial treatment (57, 72, 74–76). In addition to the community’s financial constraints, insufficient resources, lack of essential medicines, poor services, and no free laboratory tests at different levels of government health facilities made pharmacies and medicals as the most appealing choice for people (28) (Table 4).

### 3.9 Enablers to address driver of AMR: community-based interventions in human and animal health

Four community-based interventions were intervened in different settings of the community to address the drives of AMR (44, 47, 49, 77). Of them, only one was focused on animal health (77) (Table 4).

A study conducted in private schools, where households of 6–9 grade students were sampled, intervened a package of training for schoolteachers, journalists, the interaction of trained teachers and school students using key messages and the communication of key messages via radio and magazine for one year (47). The result showed improved knowledge on antibiotics and the consequences of inappropriate use of antibiotics (47). Similarly, another intervention consisted of training of community leaders and drug sellers, educational program in schools, and street theater performances, followed by discussions with mother of less than five years. (49). Following the intervention, the use of prescribed antibiotics and health post-attendance increased among under five severe acute respiratory infection cases (49). Another study showed increased knowledge, attitude, and practice score after an educational intervention among guardian of pediatrics admitted in one of the tertiary hospital (44). However, this study mentioned that despite the guardian having good knowledge and positive attitude, good practice was low (44). In case of animal health, one case–control study was conducted with livestock hygiene education as an intervention, which illustrated the improvement in animal husbandry practices of the trained household (77) (Table 4).

Furthermore, articles highlighted the importance of behavioral change in public, policymakers, prescribers, farmers, and other One Health stakeholders via community awareness program (54, 68). These articles suggested that increased use of mobile devices, social media, mobile games, and Internet in the present context could be taken as an opportunity to sensitize and make a large group of people aware of AMR from One Health perspective (54, 68) (Table 4).

## 4 Discussion

This scoping review represents the first exploration of drivers of AMR, specific to communities of Nepal. The purpose of this study was to map the existing evidence on the drivers of AMR from One Health perspective at the community level. Overall, this study showed that limited studies are conducted in the mountain region of Nepal. Additionally, the bulk of studies focus on human health, followed by limited studies on animal health, whereas no studies have yet been conducted to understand the environmental aspects of AMR that exist at the community level. Furthermore, original studies conducted in Nepal have explored the use of antibiotics and knowledge on ABR, whereas limited studies presented on wider antimicrobials use and AMR related to fungal, parasitic, or viral infections. This is an important research gap, as ongoing research by the authorship team has found that antifungal, antiprotozoal, and antiparasitic drugs are as commonly misused as antibiotics in the community (79). Recently published work demonstrated that community people use colloquial terminologies or concepts to refer to each type of antimicrobial drugs (79). Understanding such terminology prior to conducting studies would help to unveil the practices of all antimicrobial drugs, which would contribute to a more holistic understanding of AMR at the community level (79).

Our study revealed that low physician-to-patient ratio (1:1724) combined with community’s challenges (such as high out-of-pocket expenditure, inability to afford laboratory test, and long distance) compelled community people to visit the nearest and cheapest alternative for the treatment, which turns out to be registered and unregistered medicals, pharmacies, and agrovets (67). The Department of Drug Administration (DDA) under the Ministry of Health and Population is the regulatory body of Nepal to regulate the sales and distribution of drugs (including antimicrobials) and prevent their misuse (80). All types of pharmacy outlets (i.e., wholesale and retail pharmacy, medical, and agrovet) in Nepal must be registered with this department (80). Furthermore, the officially sanctioned role of authorized person to operate all types of pharmacy outlets does not encompass the diagnosis and treatment of patients (80). However, one of the studies showed that the number of unregistered pharmacy outlets was comparatively more in the rural areas (41). Reflecting ineffectiveness of policy execution, remote, and rural areas showed a greater occurrence of multidrug-resistant bacterial strains because of limited access to health posts and hospitals (63). Furthermore, drug sellers’ poor antimicrobial dispensing practices such as unnecessary use, over-prescribing (where one would be sufficient), and opting for broad-spectrum antimicrobials (where narrow spectrum could be effective) were identified in this study. A study identified these behaviors of drug sellers were driven by business motive of antibiotic companies where they provide special offers to drug sellers for selling certain amount of antibiotics (28). Another study showed that increasing the number of drugs dispensed increased the occurrence of dispensing errors and reduced the patient’s understanding about the dosing schedule (74). Hence, these poor antimicrobial dispensing practices are facilitating early development of AMR (25, 41, 48, 63, 67, 69, 74).

In case of animal health, our study identified antimicrobials that are being used for disease prevention, treatment, growth promotion, and reduction in mortality of animals such as cattle, pigs, and poultry. This depicts the clear gap between policy and practice as one study reported, and commercial poultry producers could not recall Nepal’s centralized government’s particular policies on restricting the use of antimicrobials in healthy livestock population (55). Additionally, failure to follow the recommended withdrawal period, poor hygiene and sanitation, and a lack of proper advice on scientific husbandry practices are key factors responsible for growing AMR in Nepal (26, 59, 63). The ignorance of the drug withdrawal period in animals has large-scale consequences on human health when animal products with antimicrobial residues above the permitted level are sold in the market (59, 60, 63). Moreover, transmission of resistant microbes of animals can occur to humans (or vice versa) via direct or indirect contact through environment, where hygiene plays a major role in the prevention of transmission. Hence, in the context of Nepal, insufficient infection control measures, inadequate management of hospital and medical waste, improper disposal of waste, inadequate sanitation, and hygiene were recognized as factors contributing to Nepal’s AMR problem.

In the context of limited understanding of public on antimicrobials and AMR, initial step could be raising community awareness to prevent AMR targeting both community people and service providers. The content to create community awareness on AMR could include rationale use of antimicrobial, importance of completing full course of antimicrobials, consequences of self-medication, over-use of antimicrobials, antimicrobial resistance, and how it spreads to food chain, environment, human, and animal health (59). Various creative approaches, such as diverse types of online or offline games (like puzzles), social media platforms (such as Facebook and Twitter), movies, and advertisements centered around AMR, can be employed to reach out the larger audience (68). Incorporating AMR-related subjects into school and college curriculum can be proposed to stimulate students’ evolving perspectives, encouraging their parents to get involved in addressing AMR, thus potentially fostering enduring effects (54). In addition to this, other approaches to control AMR are precise monitoring on registration status of drug sellers and rational use of the antimicrobials by the service providers. Strict implementation of the policy where prescription is required for buying and selling antimicrobials is another vital step to be taken to ensure rationale use of antimicrobials. Furthermore, there is a need for more strengthened surveillance system as it plays a crucial role in monitoring AMR and shaping policies along with its implementation and responses for preventing infections (26).

## 5 Limitation

This scoping review mapped the evidence on One Health drivers of AMR existing within the community. However, we did not find a published origin focusing on the community’s behavioral aspects contaminating environmental health that propagates AMR. Furthermore, initially, we aimed to assess potential differences across gender, geographic location (including rural/urban), ethnicity/religion, and education status of the community people. Nevertheless, the evidence found was not adequate to explore the differences within the mentioned community groups. Lastly, critical appraisals of the included articles were not performed due to the time constraint, as this was conducted as a part of formative work for the project titled “Community solutions to AMR (COSTAR)” (81).

## 6 Conclusion

Limited awareness regarding antimicrobials and antimicrobial resistance (AMR), coupled with their inappropriate usage across diverse community people in both human and animal healthcare sectors, warns about the alarming condition of AMR in Nepal. The community’s tendency to avoid formal healthcare facilities has led to heightened antimicrobial usage without prescriptions. This behavior is further exacerbated by complex social and economic ties between community people and drug sellers, accelerating the inappropriate use of antimicrobials. This situation indicates a failure in implementing established policies promoting the prudent use of antimicrobials.

Addressing this challenge necessitates different types of community engagement approaches aimed at enhancing awareness and changing the behavior concerning AMR at community level. Simultaneously, acknowledging the significant role of medical, pharmacies, and agrovets in Nepal, their involvement in enhancing knowledge is crucial. It is imperative to emphasize on their appropriate antimicrobial dispensing practices while prioritizing ‘patient education’ to promote the appropriate use of antimicrobials.

Additionally, strict enforcement of policies advocating appropriate use of antimicrobial, strong political dedication, and seamless collaboration among One Health stakeholders is pivotal in averting the escalating threat of AMR and safeguarding human health.

## Data availability statement

The original contributions presented in the study are included in the article/Supplementary material, further inquiries can be directed to the corresponding author.

## Author contributions

AP: Conceptualization, Data curation, Formal analysis, Investigation, Methodology, Writing – original draft, Writing – review & editing. JM: Conceptualization, Investigation, Supervision, Validation, Writing – review & editing. NK: Data curation, Methodology, Writing – review & editing, Investigation. AA: Conceptualization, Supervision, Validation, Writing – review & editing. SL: Conceptualization, Writing – review & editing. RK: Conceptualization, Funding acquisition, Supervision, Writing – review & editing. SB: Conceptualization, Funding acquisition, Supervision, Writing – review & editing.
