# Supplementary material for: Drivers of antimicrobial resistance within the communities of Nepal from One Health perspective: a scoping review
Source: Front Public Health. 2024 Apr 19;12:1384779. doi: 10.3389/fpubh.2024.1384779 (PMC11066241; doi:10.3389/fpubh.2024.1384779)
Supplement: Supplementary file 1 [file Data_Sheet_1.docx]

**Supplementary material 1**

**Key words used:**

1. **Antimicrobials**

- anti-biotic*  OR  anti-microbial*  OR  anti-infective*  OR  anti-viral*  OR  anti-protozoal*  OR  anti-parasitic*  OR  anti-bacterial*  OR  anti-fungal*  OR  anti-helminth*  OR  anti-malarial*
- antibiotic*  OR  antimicrobial*  OR  antiinfective*  OR  antiviral*  OR  antiprotozoal*  OR   antiparasitic*  OR  antibacterial*  OR  antifungal*  OR  antihelminth*  OR  antimalarial*

1. (drug* OR multidrug* OR multi-drug*) resistance
2. Nepal

**Table 1 Summary of databases searched**

| **Database searched**  **(limit: 2000-2023)** | **Initial search** | | | **Updated search** | | |
| --- | --- | --- | --- | --- | --- | --- |
|  | **Date searched** | **Searched by** | **Hits** | **Date searched** | **Searched by** | **Hits** |
| PubMed | 2021-12-3 | AP | 1180 | 2023-02-27 | NK | 1299 |
| CINAHL | 2021-12-3 | AP | 321 | 2023-02-28 | NK | 360 |
| SCOPUS | 2021-12-3 | AP | 1520 | 2023-02-27 | NK | 1816 |
| Global index medicus | 2021-12-3 | AP | `164 | 2023-02-28 | NK | 166 |
| Google scholar | 2021-12-6 | AP | 993 | 2023-02-28 | NK | 1000 |
| HINARI-SUMON search | 2021-12-7 | AP | 379 | - | - | - |
| Embase (Ovid) | 2021-11-25 | NK | 1893 | 2023-02-27 | NK | 2274 |
| Global Health (Ovid) | 2021-11-25 | NK | 1229 | 2023-02-27 | NK | 1435 |
| CAB Abstracts (Ovid) | 2021-11-25 | NK | 849 | 2023-02-27 | NK | 1056 |
| Web of Science | 2021-11-25 | NK | 1203 | 2023-02-27 | NK | 1286 |
| **Total hits** |  | | **9567** |  | | **11071** |
| **After de-duplication** |  | | **3694** |  | | **749** |

**Details of the search strategy (initial search) for each database are follows:**

**Table 2 PubMed**

| **Search #** | **Search concept** | **Search query** | **Results** |
| --- | --- | --- | --- |
| 1 | Antimicrobial | anti-biotic* [Title/Abstract] OR anti-microbial* [Title/Abstract] OR anti-infective* [Title/Abstract] OR anti-viral* [Title/Abstract] OR anti-protozoal* [Title/Abstract] OR anti-parasitic* [Title/Abstract] OR anti-bacterial*[Title/Abstract] OR anti-fungal*[Title/Abstract] OR anti-helminth* [Title/Abstract] OR anti-malarial* [Title/Abstract] | 31,488 |
| 2 |  | antibiotic* [Title/Abstract] OR antimicrobial* [Title/Abstract] OR antiinfective* [Title/Abstract] OR antiviral* [Title/Abstract] OR antiprotozoal* [Title/Abstract] OR antiparasitic*[Title/Abstract] OR antibacterial*[Title/Abstract] OR antifungal*[Title/Abstract] OR antihelminth* [Title/Abstract] OR antimalarial* [Title/Abstract] | 750,511 |
| 3 |  | "Anti-Infective Agents"[Mesh] OR "Antimicrobial Stewardship"[Mesh] | 769,484 |
| 4 | Drug/multidrug resistance | ("drug* resistan*"[Text Word]) OR ("multidrug* resistan*"[Text Word]) OR ("multi-drug* resistan*"[Text Word]) | 345,185 |
| 5 |  | "Drug Resistance"[Mesh] OR "Drug Resistance, Multiple"[Mesh] OR "Drug Resistance, Microbial"[Mesh] | 355,030 |
| 6 | Antimicrobial OR Drug/multidrug resistance | 1 OR 2 OR 3 OR 4 OR 5 | 1,410,154 |
| 7 | Nepal | Nepal*[Text Word] | 14,354 |
| 8 |  | "Nepal"[Mesh] | 9,642 |
| 9 |  | 7 OR 8 | 14,354 |
| 10 | Final search | 6 AND 9 | 1268 |
| 11 | With filter (2000-present) | 6 AND 9 | 1180 |

**Table 3 CINAHL**

**Limiters/Expanders:** Expanders - Apply equivalent subjects
 Search modes - Boolean/Phrase

The final search was limited to 2000-2021

| **Search #** | **Concepts** | **Query** | **Results** |
| --- | --- | --- | --- |
| S1 | Anti-microbial | TI ( anti-biotic* OR anti-microbial* OR anti-infective* OR anti-viral* OR anti-protozoal* OR anti-parasitic* OR anti-bacterial* OR anti-fungal* OR anti-helminth* OR anti-malarial* ) OR AB ( anti-biotic* OR anti-microbial* OR anti-infective* OR anti-viral* OR anti-protozoal* OR anti-parasitic* OR anti-bacterial* OR anti-fungal* OR anti-helminth* OR anti-malarial* ) | 2686 |
| S2 |  | TI ( antibiotic* OR antimicrobial* OR antiinfective* OR antiviral* OR antiprotozoal* OR antiparasitic* OR antibacterial* OR antifungal* OR antihelminth* OR antimalarial* ) OR AB ( antibiotic* OR antimicrobial* OR antiinfective* OR antiviral* OR antiprotozoal* OR antiparasitic* OR antibacterial* OR antifungal* OR antihelminth* OR antimalarial* ) | 90786 |
| S3 |  | (MH "Antimicrobial Stewardship") OR (MH "Antiinfective Agents+") | 172298 |
| S4 | Drug/Multidrug resistance | TI ( "drug* resistan*" OR "multi-drug* resistan*" OR "multidrug* resistan*" ) OR AB ( "drug* resistan*" OR "multi-drug* resistan*" OR "multidrug* resistan*" ) | 16736 |
| S5 |  | (MH "Drug Resistance+") OR (MH "Drug Resistance, Microbial+") OR (MH "Drug Resistance, Multiple") | 59686 |
| S6 | Antimicrobial OR Drug/Multidrug resistance | S1 OR S2 OR S3 OR S4 OR S5 | 254983 |
| S7 | Nepal | TX Nepal* | 5542 |
| S8 |  | (MH "Nepal") | 3242 |
| S9 |  | S7 OR S8 | 5542 |
| S10 | Final search | S6 AND S9 | 331 |
| S11 | Final search | S6 AND S9 (date: 2000-present) | 321 |

**Table 4 SCOPUS**

| **Search #** | **Concept** | **Search query** | **Result** |
| --- | --- | --- | --- |
| 1 | antimicrobials | TITLE-ABS ( anti-biotic*  OR  anti-microbial*  OR  anti-infective*  OR  anti-viral*  OR  anti-protozoal*  OR  anti-parasitic*  OR  anti-bacterial*  OR  anti-fungal*  OR  anti-helminth*  OR  anti-malarial* )  OR  TITLE-ABS ( antibiotic*  OR  antimicrobial*  OR  antiinfective*  OR  antiviral*  OR  antiprotozoal*  OR  antiparasitic*  OR  antibacterial*  OR  antifungal*  OR  antihelminth*  OR  antimalarial* ) | 1,011,472 |
| 2 | Drug/multi-drug resistance | (TITLE-ABS-KEY ("drug resistan*) OR TITLE-ABS-KEY ("multi-drug* resistan*") OR TITLE-ABS-KEY ("multidrug* resistan*") ) | 391,536 |
| 3 | Antimicrobial OR Drug/Multidrug resistance | 1 OR 2 | 1,281,602 |
| 4 | Nepal | TITLE-ABS-KEY (nepal*) OR AFFILCOUNTRY (nepal*) | 43,127 |
| 5 | Final search | 3 AND 4 | 1,600 |
| 6 | Final search | 3 AND 4 (2000-present) | 1520 |

**Global Index Medicus**

1. (tw:(resistan* AND (drug* OR multidrug* OR Multi-drug))) OR (tw:(antibiotic* OR antimicrobial* OR anti-infective* OR antiviral* OR antiprotozoal* OR anti-parasitic* OR antibacterial* OR antifungal* OR antihelminth* OR antimalarial* )) AND (tw:(nepal)) = 108

Considering more numbers of articles obtained, search strategy was broken down into two parts a) and b) in the final search

1. (tw:(antibiotic* OR antimicrobial* OR anti-infective* OR antiviral* OR antiprotozoal* OR anti-parasitic* OR antibacterial* OR antifungal* OR antihelminth* OR antimalarial* )) AND (tw:(nepal*)) = 108
2. (tw:(resistan* AND (drug* OR multidrug* OR Multi-drug))) AND (tw:(nepal*)) = 56

**Google scholar**

(antimicrobial* OR anti-infective* OR antibiotic* OR "multi-drug* resistance*" OR "drug* resistance*") AND Nepal* = 993 [With date limit 2000-2021]

**HINARI (SUMMON)**

((TitleCombined:(antibiotic* OR antimicrobial* OR anti-infective* OR antiviral* OR antiprotozoal* OR anti-parasitic* OR antibacterial* OR antifungal* OR antihelminth* OR antimalarial*)) OR (Abstract:(antibiotic*  OR  antimicrobial*  OR  anti-infective*  OR  antiviral*  OR  antiprotozoal*  OR  anti-parasitic*  OR  antibacterial*  OR  antifungal*  OR  antihelminth*  OR  antimalarial*)) OR (TitleCombined:("multi-drug* OR resistance*\\" OR \\"drug* OR resistance*")) OR (Abstract:("multi-drug* resistance*" OR "drug* resistance*"))) AND ((TitleCombined:(Nepal*)) OR (Abstract:(Nepal*)))

= 379 [With date limit 2000-2021]

**Table 5 CAB Abstracts (Ovid) <2000 to 2021 Week 47>**

| **Search #** | **Search query** | **Results** |
| --- | --- | --- |
| 1 | nepal/ | 12788 |
| 2 | Nepal*.ti,ab,in,ad,hw. | 16169 |
| 3 | 1 or 2 | 16169 |
| 4 | exp antiinfective agent/ | 186210 |
| 5 | (antibiotic* or antimicrobial* or antiinfective* or antiviral* or antiprotozoal* or antiparasitic* or antibacterial* or antifungal* or antihelminth* or antimalarial*).tw,hw. | 352839 |
| 6 | (anti-biotic* or anti-microbial* or anti-infective* or anti-viral* or anti-protozoal* or anti-parasitic* or anti-bacterial* or anti-fungal* or anti-helminth* or anti-malarial*).tw,hw. | 99077 |
| 7 | exp drug resistance/ | 61723 |
| 8 | ((multidrug* or multi-drug* or drug*) adj3 resistan*).tw,hw. | 71680 |
| 9 | or/4-8 | 443912 |
| 10 | 3 and 9 | 849 |

**Table 6 Embase (Ovid) <1996 to 2021 Week 46>**

| **Search #** | **Search query** | **Results** |
| --- | --- | --- |
| 1 | Nepal*.ti,ab,in,ad,kf. | 22678 |
| 2 | Nepal/ | 12827 |
| 3 | 1 or 2 | 23633 |
| 4 | *antiinfective agent/ or exp *antibiotic agent/ or exp *antifungal agent/ or exp *antimycobacterial agent/ or exp *antispirochetal agent/ | 478665 |
| 5 | antibiotic* or antimicrobial* or antiinfective* or antiviral* or antiprotozoal* or antiparasitic* or antibacterial* or antifungal* or antihelminth* or antimalarial*).tw,kf. | 809900 |
| 6 | (anti-biotic* or anti-microbial* or anti-infective* or anti-viral* or anti-protozoal* or anti-parasitic* or anti-bacterial* or anti-fungal* or anti-helminth* or anti-malarial*).tw,kf. | 45264 |
| 7 | exp antibiotic resistance/ | 161408 |
| 8 | antifungal resistance/ or antimalarial drug resistance/ or antiviral resistance/ or cross resistance/ or extensive drug resistance/ or multidrug resistance/ | 68989 |
| 9 | ((multidrug* or multi-drug* or drug*) adj3 resistan*).tw,kf. | 198164 |
| 10 | antimicrobial stewardship/ | 7016 |
| 11 | or/4-10 | 1260296 |
| 12 | 3 and 11 | 1938 |
| 13 | limit 12 to yr="2000 -Current" | 1893 |

**Table 7 Global Health (Ovid) <1973 to 2021 Week 47>**

| **Search #** | **Search query** | **Results** |
| --- | --- | --- |
| 1 | nepal/ | 7891 |
| 2 | Nepal*.ti,ab,in,ad,hw. | 9591 |
| 3 | 1 or 2 | 9591 |
| 4 | exp antiinfective agent/ | 301180 |
| 5 | (antibiotic* or antimicrobial* or antiinfective* or antiviral* or antiprotozoal* or antiparasitic* or antibacterial* or antifungal* or antihelminth* or antimalarial*).tw,hw. | 464824 |
| 6 | (anti-biotic* or anti-microbial* or anti-infective* or anti-viral* or anti-protozoal* or anti-parasitic* or anti-bacterial* or anti-fungal* or anti-helminth* or anti-malarial*).tw,hw. | 85277 |
| 7 | exp drug resistance/ | 123601 |
| 8 | ((multidrug* or multi-drug* or drug*) adj3 resistan*).tw,hw. | 137511 |
| 9 | or/4-8 | 559080 |
| 10 | 3 and 9 | 1329 |
| 11 | limit 10 to yr="2000-current" | 1229 |

**Web of Science Core collection (Clarivate)**

The following databases were searched simultaneously from the Core Collection

- Science Citation Index Expanded (SCI-EXPANDED)--1900-present
- Social Sciences Citation Index (SSCI)--1900-present
- Arts & Humanities Citation Index (AHCI)--1975-present
- Conference Proceedings Citation Index – Science (CPCI-S)--1990-present
- Conference Proceedings Citation Index – Social Science & Humanities (CPCI-SSH)--1990-present
- Emerging Sources Citation Index (ESCI)--2015-present

*Data updated 2021-11-23*

**Table 8 Web of Science**

| **Search #** | **Search query** | **Results** |
| --- | --- | --- |
| 1 | antibiotic* OR antimicrobial* OR antiinfective* OR antiviral* OR antiprotozoal* OR antiparasitic* OR antibacterial* OR antifungal* OR antihelminth* OR antimalarial* (Topic) | 880,446 |
| 2 | anti-biotic* OR anti-microbial* OR anti-infective* OR anti-viral* OR anti-protozoal* OR anti-parasitic* OR anti-bacterial* OR anti-fungal* OR anti-helminth* OR anti-malarial (Topic) | 40,709 |
| 3 | (drug* OR multidrug* OR "multi drug*") NEAR/2 resistan* (Topic) | 195,398 |
| 4 | #1 OR #2 OR #3 | 1,039,092 |
| 5 | TS=(Nepal*) OR AD=(Nepal*) | 34,423 |
| 6 | #4 AND #5 | 1,239 |
| 7 | #4 AND #5 (Date limit used 2000-01-01 to 2021-12-31) | 1,203 |
